# Physiological Striae Atrophicae of Adolescence with Involvement of the Axillae and Proximal Arms

**DOI:** 10.1155/2017/7678086

**Published:** 2017-05-16

**Authors:** Alexander K. C. Leung, Benjamin Barankin

**Affiliations:** ^1^Department of Pediatrics, The University of Calgary, Calgary, AB, Canada; ^2^The Alberta Children's Hospital, Calgary, AB, Canada T2M 0H5; ^3^Toronto Dermatology Centre, Toronto, ON, Canada M3H 5Y8

## Abstract

We report a 16-year-old adolescent male with multiple violaceous, atrophic, vertical linear striae isolated to the axillae and proximal arms of approximately one-year duration. In the past two years, he indulged in heavy weight-lifting. He experienced a growth spurt over the past few years. The patient was otherwise in good health and was not on any medications. Physiological striae atrophicae of adolescence where the striae were restricted to the axillae and proximal arms have very rarely been reported.

## 1. Introduction

Physiological striae atrophicae of adolescence, also known as physiological striae atrophicae of puberty, occur mainly in healthy, nonobese individuals at around puberty in association with the adolescent growth spurt. We describe a 16-year-old adolescent male with physiological striae atrophicae of adolescence presenting with multiple purplish, atrophic, vertical linear striae isolated to the axillae and proximal arms. To our knowledge, physiological striae atrophicae of adolescence where the striae were restricted to the axillae and proximal arms have very rarely been reported.

## 2. Case Report

A 16-year-old adolescent male presented with multiple linear striae affecting both axillae and proximal arms. The striae were first noted a year ago. For the past two years, he indulged in heavy weight-lifting in a fitness center. He spent at least one hour per day in heavy weight-lifting, several days per week. The patient had quite a rapid growth spurt over the past few years. He grew 10 to 12 cm and gained 5 to 6 kg per year in the past two years. The patient noted that the deltoid areas became larger with the growth spurt and weight-lifting. He was in good health and was not on any medications. There was no family history of similar skin lesions.

**Figure 1:**
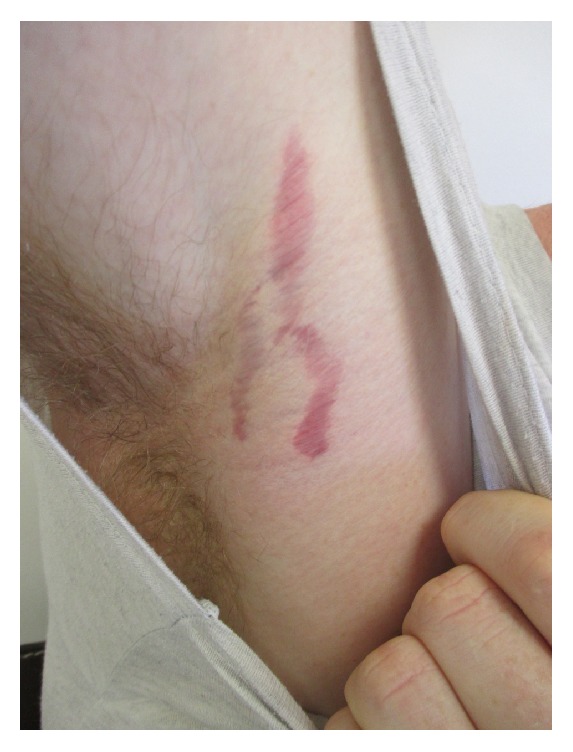
Violaceous, atrophic, linear striae in the right axilla and proximal arm.

On examination, he was alert and not in distress. His height was 165 cm and weight 57 kg with a body mass index (BMI) of 20.9 kg/m^2^. His heart rate was 70 beats per minute and blood pressure 105/75 mm Hg. Multiple violaceous, atrophic, vertical linear striae were noted affecting the axillae and proximal arms (Figure 1). There were no striae elsewhere on the body. His pubic hair was of adult quality but did not spread to the junction of the medial thigh with perineum (Tanner stage 4). Hair was noted in both axillae. The long axis of the testes measured 4 cm. The penile length was 5.5 cm when flaccid. Acne vulgaris was noted on his face and forehead. All other physical findings were normal.

## 3. Discussion

Transverse linear striae on the back in adolescence were first described in 1917 by Weber who reported an 18-year-old tailor with this condition [1]. Weber ascribed the condition to the rapid growth at puberty: “the growth of the skin apparently could not keep up with the growth of the muscles, subcutaneous tissues and long bones.” The term “idiopathic striae atrophicae of puberty” was coined by Weber in 1935 to describe onset of this condition around puberty without an identifiable underlying cause [2].

Typically, physiological striae atrophicae of adolescence occur in nonobese, healthy adolescents undergoing rapid linear growth at the time of puberty [3]. There is a male predominance possibly because males have a faster growth rate than females at around puberty [3]. The peak age of onset is usually between 14 and 20 years of age in males and 10 and 16 years of age in females [4].

Characteristically, physiological striae atrophicae of adolescence present as violaceous or red, horizontal, linear streaks (striae rubra) in the lumbar area, giving rise to a “wash board” appearance [3, 5]. Over time, the color fades and the lesions become atrophic and silvery (striae alba) [3]. The striae are usually 1 to 10 mm wide and several cm long, with the long axis perpendicular to the direction of skin tension [6].

The occurrence of physiological striae atrophicae of adolescence other than in the lumbar area is rare [7]. In 2013, Leung and Barankin reported a 13-year-old boy with physiological striae atrophicae of adolescence strictly confined to the upper thoracic area [8]. According to the authors, this finding had not been reported previously. Herein, we report a 16-year-old adolescent male with physiological striae atrophicae of adolescence affecting the axillary areas and proximal arms. To the best of our knowledge, the occurrence of physiological striae atrophicae of adolescence isolated to the axillae and proximal arms has very rarely been reported.

The association of physiological striae atrophicae of adolescence and weight-lifting has been reported only occasionally in the past [7]. In 1964, Shelley and Cohen described a 16-year-old male with a linear atrophic stria running transversely over a lower thoracic vertebra [7]. The patient had been weight-lifting and performing trampoline exercises during the preceding few years. The authors attributed the stria to a dermal tear resulting from repetitive weight-lifting. Our patient was also a weight-lifter. In the present case, it is possible that repetitive weight-lifting and rapid growth can result in chronic mechanical shearing and stretching of the skin leading to rupture of dermal collagen fibres. Hormonal factors (e.g., hypercortisolemia), innate structural disturbance of the integument, and genetic factors may also be operative. Reduced levels of genes encoding fibronectin and collagen may lead to thinning of connective tissue with resulting susceptibility to striae atrophicae [9].

We and other authors believe that physiological striae atrophicae of adolescence and striae distensae (stretch marks) are separate disease entities [3, 4, 8]. On the other hand, some authors have suggested that physiological striae atrophicae of adolescence are a type of striae distensae. Further research, including histologic or biochemical analysis, may provide more insight in this area. In striae distensae, the striae are typically located on the lateral thighs, lower abdomen, buttocks, and, in the females, the breasts [4]. Atypical locations include the groins, outer aspect of the upper arms, shoulders, and legs [4, 10]. Involvement of the axillae has rarely been reported [10–12]. Very rarely, the condition can be generalized [13]. There is a female predominance in striae distensae [5]. Risk factors for striae distensae include obesity, rapid weight gain, pregnancy (especially with multiple pregnancy and polyhydramnios), prolonged use of topical or systemic corticosteroids, positive family history of striae distensae, Cushing syndrome, prolonged adrenocorticotropic hormone (ACTH) therapy, prolonged use of oral contraceptives, human-immunodeficiency virus (HIV) therapy, vitamin D deficiency, and Marfan syndrome [14–16]. Other conditions associated with striae distensae include augmentation mammoplasty, intense slimming diets, anorexia nervosa, excessive use of marijuana, chemotherapy, chronic liver disease, rheumatic fever, typhoid fever, cardiac surgery, and organ transplantation [14, 17].

Physiological striae of adolescence have been mistaken in the past for bruises resulting from nonaccidental injury [6, 9, 18–21]. Misdiagnosis is particularly likely to occur in a nonobese, comatose adolescent with striae in uncommon locations such as the upper back and axilla. Increased awareness of this condition is crucial so that false accusations of child abuse are not made.

## 4. Conclusion

Physiological striae atrophicae of adolescence typically occur in the lumbar area. The occurrence of physiological striae atrophicae of adolescence isolated to both axillae and proximal arms has very rarely been reported. Awareness of this condition is important so that false accusations of child abuse are not made.
